# The Food of the People

**Published:** 1906-11-10

**Authors:** 


### THE FOOD OF THE PEOPLE.

Sir Frederick Treves spoke well and to the point at the meeting of the National Health Society, and rightly maintained that the food supply of the people should be ample and good. Sir Frederick Treves maintains with conviction that the present infantile mortality is to a very large extent preventable, and that it is due to food. He further states that it could be reduced to a degree of which the present generation has probably no conception, if only the infants of this country could be supplied with pure milk in adequate quantities. The remedy lies in the first place in the direction of (1) securing an improvement in the quality of the milk supply itself, and (2) the setting up of a standard which will improve the conditions under which the milk trade is at present carried on. Some municipalities have shown wisdom, in organising methods, to enable the poor to obtain adequate supplies of the best milk for their children. This duty devolves upon every municipality where the interests of the ratepayers and the nation are, as they ought to be, the first consideration with these authorities.

In the matter of food the ignorance of the people as a whole, and their indifference in regard to what they eat, constitute the great difficulties in securing adequate health protection in this respect. The foreign and American practice of teaching children simple rules of health, which include a knowledge of what to avoid, should be adopted in all British schools. Nothing more remarkable has occurred in the world's history than the effects of such teaching upon leprosy in Norway. With better knowledge the ravages of many diseases could be diminished, and the happiness of the people greatly increased. The present system of food inspection is inadequate and largely valueless. All such inspections should take place at the source, and not merely in the market or the kitchen. Here, and this is a matter which demands the immediate attention of the government, legislation can do much to provide greater security for the people.

In the poorer districts of all large cities there is need for a well organised system of eating houses and restaurants, where the best food of the purest kind can be guaranteed to every customer. Several public spirited citizens, and some keen men of business, are doing much useful service in this direction. Each municipality, through its health department, might usefully institute a system of periodical inspection, and the issue of certificates of merit to every such establishment, which was found to afford the consumer the fullest guarantees, that all the food supplied was of the purest and best description. The municipal reformers, through their recent victory, have a unique opportunity, to make an enduring reputation for themselves, by securing an adequate and wholesome supply of food, for the people, throughout London.

